# Comparison of Remimazolam-Based Monitored Anesthesia Care and Inhalation-Based General Anesthesia in Transurethral Resection of Bladder Tumor: A Randomized-Controlled Trial

**DOI:** 10.3390/cancers17050848

**Published:** 2025-02-28

**Authors:** Jin Sun Cho, Won Sik Ham, Bahn Lee, Hyun Il Kim, Jin Ha Park

**Affiliations:** 1 Department of Anesthesiology and Pain Medicine, Yonsei University College of Medicine, Seoul 03722, Republic of Korea; chjs0214@yuhs.ac (J.S.C.); bahnlee@yuhs.ac (B.L.); choco8926@yuhs.ac (H.I.K.); 2 Anesthesia and Pain Research Institute, Yonsei University College of Medicine, Seoul 03722, Republic of Korea; 3 Department of Urology, Urological Cancer Center, Severance Hospital, Yonsei University College of Medicine, Seoul 03722, Republic of Korea; uroham@yuhs.ac

**Keywords:** analgesia nociception index, general anesthesia, inhalation, monitored anesthesia care, transurethral resection of bladder tumor, remimazolam

## Abstract

Background/Objectives: Transurethral resection of bladder tumor (TURBT) is commonly performed in elderly patients and often requires repeated procedures. A high proportion of non-procedural time in TURBT affects operating room utilization, highlighting the need for a safe and efficient anesthesia method. This study aimed to compare remimazolam-based monitored anesthesia care (MAC) guided by analgesia nociception index (ANI) monitoring to inhalation-based general anesthesia (GA) for TURBT, focusing on induction and emergence time (IAET). Methods: Forty-six patients who underwent TURBT were randomly assigned into either the MAC group or GA group. The primary outcome was the IAET. The secondary outcomes included hospital and anesthesia charges, safety (intraoperative hypotension and desaturation), and feasibility (surgeon and patient satisfaction) of both anesthetic methods. Results: The MAC group demonstrated a significantly shorter IAET (14 vs. 25 min, *p* < 0.001) and lower anesthesia cost (USD 152 vs. USD 195, *p* < 0.001). The MAC group showed better hemodynamic stability with a lower incidence of hypotension (29% vs. 73%, *p* = 0.004). Seven patients (33%) in the MAC group experienced intraoperative desaturation; all patients recovered without complications through the jaw-thrust maneuver. Patient satisfaction was equally high in both groups, and surgeon satisfaction, though slightly lower with MAC (71% vs. 100% rating “excellent”, *p* = 0.009), remained acceptable. Conclusions: Remimazolam-based MAC, guided by ANI monitoring, offers significant advantages including shorter IAET, reduced costs, and improved safety for TURBT, particularly in patients with small tumors. These findings support MAC as a promising alternative to GA for TURBT, as it enhances perioperative outcomes and operating room efficiency.

## Simple Summary

Bladder cancer is a common cancer, and transurethral resection of bladder tumor (TURBT) is the cornerstone of bladder cancer diagnosis and treatment. TURBT is a high-throughput procedure, with non-procedure time accounting for 50% of total operation time, highlighting the importance of anesthesia-controlled time efficiency. This randomized-controlled study aimed to compare the remimazolam-based monitored anesthesia care (MAC) to the inhalation-based general anesthesia (GA) in patients undergoing TURBT. Patients who underwent TURBT with remimazolam-based MAC experienced significantly reduced induction and emergence times compared to those who received inhalation-based GA. Remimazolam-based MAC also provided better hemodynamic stability, with a lower incidence of hypotension and reduced anesthesia costs. Remimazolam-based MAC may be a promising alternative to inhalation-based GA in patients undergoing TURBT, specifically for small bladder tumors.

## 1. Introduction

Transurethral resection of bladder tumor (TURBT) is the cornerstone of bladder cancer diagnosis and treatment [1]. Bladder tumor is among the ten most common cancers worldwide, making it one of the costliest diseases to manage [2,3,4]. Bladder tumors have recurrence rates of 15–70%, often requiring repeated TURBT [5,6]. With increases in the elderly population and bladder tumor incidence, the demand for repeated TURBT is expected to increase, adding to the healthcare burden.

The operating room (OR) accounts for 35–40% of hospital costs, and with surgical care accounting for approximately one-third of healthcare spending [7,8], OR performance and efficiency can be optimized through improved utilization and time management without using additional resources [7]. In TURBT, with its relatively short operation time of 18–45 min [9,10], non-procedural time constitutes a significant proportion of total operation time, highlighting the importance of anesthesia-controlled time efficiency.

TURBT is often performed under general anesthesia (GA) because of its short duration. However, given the median age at diagnosis of 73 years [6], mechanical ventilation during GA may increase the risk of pulmonary complications, potentially affecting prognosis. Therefore, there is an emerging need for anesthesia methods that not only avoid mechanical ventilation to prevent a decline in pulmonary function but also optimize anesthesia-controlled time efficiency.

Monitored anesthesia care (MAC) is a viable option for short procedures, allowing spontaneous breathing. Recent studies have demonstrated its effectiveness in third-molar extraction, resectoscopy, atrial fibrillation ablation, and endoscopic retrograde cholangiopancreatography [11,12,13,14,15]. Remimazolam, an ultra-short-acting benzodiazepine, allows a rapid onset and short recovery, making it suitable for MAC [16]. Given the short operative time and minimal pain, remimazolam-based MAC is expected to be effective during TURBT. Additionally, monitoring the analgesia nociception index (ANI), derived from heart rate variability, helps assess the levels of acute pain, thereby aiding in adjusting anesthesia and analgesic dosages [17].

In this study, we aim to compare the induction and emergence time (IAET) of remimazolam-based MAC guided by analgesia nociception index (ANI) monitoring to that of inhalation-based GA in patients undergoing TURBT.

## 2. Materials and Methods

### 2.1. Patients

This study was conducted at Severance Hospital, Yonsei University Health System, Seoul, South Korea, approved by our institutional review board (4-2023-1206), and registered on 22 January 2024, at http://www.ClinicalTrials.gov (accessed on 25 February 2025) (NCT06217055). Written consent was obtained from all patients.

Between February 2024 and October 2024, 46 patients aged ≥20 years scheduled for elective TURBT were randomly assigned in a 1:1 ratio to either the remimazolam-based MAC (MAC) group or the inhalation-based GA (GA) group using a computer-generated number table. Blinding was not conducted because sevoflurane administration requires a vaporizer. Patients were excluded if they met at least one of the following criteria preoperatively: (1) American Society of Anesthesiologists (ASA) physical status ≥IV; (2) emergency surgery; (3) history of hypersensitivity to sevoflurane, propofol, and remimazolam; (4) galactose intolerance; (5) patient refusal; and (6) patients expected to require muscle relaxation due to the need for an obturator nerve block during surgery according to the location or size of the tumor.

### 2.2. Intervention

On arrival in the OR, patients were monitored with electrocardiography (ECG), pulse oximetry (SpO_2_), non-invasive blood pressure, and patient state index (PSI) using a SedLine^®^ electroencephalograph sensor (Masimo Corp., Irvine, CA, USA). ANI monitoring was also applied to all patients. In the MAC group, supplemental oxygen (6 L/min) was administered through a facial mask. Remimazolam was administered at a rate of 6 mg/kg/h until the loss of consciousness, defined as a Modified Observer’s Alertness/Sedation (MOAA/S) score ≤ 2. Following loss of consciousness, remimazolam was administered at a maintenance dose of 1 mg/kg/h [11] and adjusted to maintain anesthetic depth at a PSI value of 30–50. Remifentanil was administered for analgesia using a target-controlled infusion at an effect-site concentration of 3.0 ng/mL and was adjusted to maintain ANI values in the range of 50–70. If the SpO_2_ was below 92%, the airway was opened simultaneously using the jaw-thrust maneuver. If the SpO_2_ remained below 92%, MAC was considered a failure, and anesthesia was converted to GA with laryngeal mask airway (LMA) insertion or endotracheal intubation. If the patient moved despite appropriate PSI and ANI scores, MAC was considered failed, and the anesthesia was changed to inhalation GA with the administration of a muscle relaxant. At the end of surgery, remimazolam and remifentanil were discontinued. If awakening, defined as a MOAA/S score ≥ 3, was not observed after 15 min from the discontinuation of remimazolam administration, then 0.2 mg flumazenil was given.

In the GA group, anesthesia was induced with propofol 1–2 mg/kg and maintained with 1–2 minimum alveolar concentrations of sevoflurane. Remifentanil was administered using the same methods in the MAC group, as aforementioned. Following loss of consciousness, LMA insertion or endotracheal intubation was performed after administration of intravenous 0.3–0.6 mg/kg rocuronium. Anesthesia and analgesia depths were adjusted to maintain PSI and ANI scores at 30–50 and 50–70, respectively. Sevoflurane and remifentanil were discontinued at the end of the surgery. The LMA or endotracheal tube was removed after the patients were awake as defined as a MOAA/S score ≥ 3.

All patients were transferred to the postoperative care unit (PACU) after awakening from the anesthesia.

### 2.3. Data Collection

The primary outcome was the IAET, which was calculated by excluding the operation time from the total anesthesia time. The secondary outcomes included hospital and anesthesia charges, as well as the safety and feasibility of the two anesthetic methods. Data of hospital and anesthesia costs were extracted from electronic medical records one month after surgery. Safety was assessed based on the occurrence of intraoperative hypotension and desaturation, the intensity of postoperative pain, and postoperative nausea and vomiting (PONV). Intraoperative hypotension was defined as a reduction in mean blood pressure below 20% of the preanesthetic value for at least 10 min, and intraoperative desaturation was defined as a SpO_2_ below 92%. The intensities of postoperative pain and PONV were measured using a numerical rating scale, where 0 denoted no pain or nausea and 10 denoted the worst pain or nausea experienced. Feasibility, evaluated using surgeon and patient satisfaction scores, was measured at the end of surgery and at the time of discharge from the PACU after full recovery, respectively. Both scores were rated using a five-point scale as described in a previous study [18]: very poor (1), poor (2), satisfactory (3), good (4), and excellent (5).

Preoperative variables included demographics, medical history, and medication. Intraoperative variables included IAET, anesthesia time, operation time, loss of consciousness time, awake time, and recovery time. The loss of consciousness time was defined as the interval between the administration of remimazolam or propofol and loss of consciousness. The awake time was defined as the time interval between the cessation of remimazolam or sevoflurane administration and patient awakening. Total doses of remimazolam, propofol, and remifentanil were recorded. The ANI, PSI, mean blood pressure, heart rate, and SpO_2_ were recorded at six different time points: before induction, immediately after induction, at the beginning of surgery, at the end of surgery, at the end of anesthesia, and upon arrival at the PACU. The MOAA/S score was measured upon arrival at the PACU. The incidences of intraoperative hypotension, desaturation, and failure of remimazolam-based MAC were recorded. Postoperative variables included the intensity of postoperative pain and PONV, which were measured upon arrival at the PACU and 6h postoperatively. The costs of anesthesia, surgery, and admission days were recorded.

### 2.4. Sample Size Calculation

The sample size was calculated based on the primary outcome. This study was powered to detect a 30% decrease in the IAET. To our knowledge, there were no previous studies evaluating the use of MAC for TURBT nor any studies reporting IAET. In addition, there is no generally accepted definition of the superiority of anesthesia methods regarding IAET. Therefore, we based our sample size calculation on preliminary data from patients who underwent GA for TURBT. We analyzed the IAET of patients who underwent TURBT under GA in our institution, calculating both the mean and standard deviation (SD) of these times. We assumed that a 30% reduction in mean IAET would be a clinically relevant improvement in anesthesia efficiency and patient recovery. The mean ± SD of IAET for TURBT performed under GA in 2022 at our institution was 27 ± 8.7 min. Twenty patients were required in each group to obtain a power of 80%, with an alpha of 0.05. Considering a 10% dropout rate, we decided to enroll 23 patients in each group.

### 2.5. Statistical Analysis

Continuous variables are shown as median (interquartile range). Dichotomous variables are expressed as the number of patients (percentage). Continuous variables were compared using the Mann–Whitney *U* test. Dichotomous variables were compared using the Fisher’s exact test. For repeated-measure variables, data were analyzed according to non-parametric tests, using a Friedman test followed by Wilcoxon signed-rank tests to compare intragroup differences. Post hoc analyses with Bonferroni correction were performed to identify group differences at each time point. A *p* < 0.05 was considered statistically significant. Statistical analyses were performed using SPSS version 28 (IBM Corp, Armonk, NY, USA) and GraphPad Prism version 5 (GraphPad Software, San Diego, CA, USA).

## 3. Results

**Figure 1:**
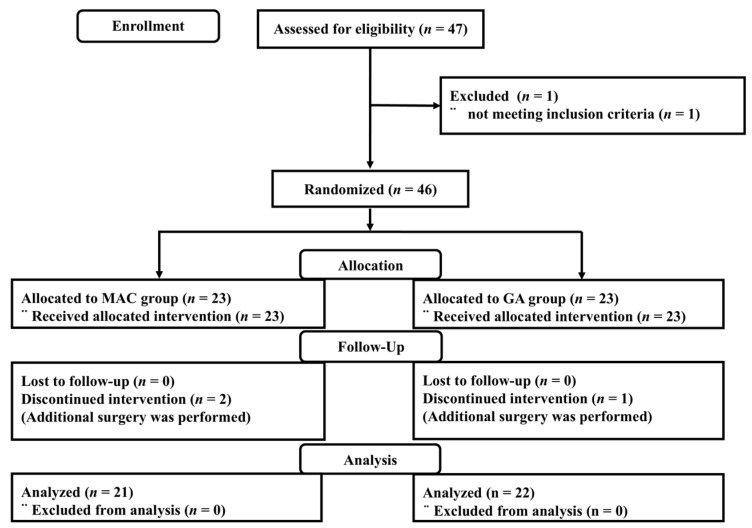
Flowchart of the study population. GA, general anesthesia; MAC, monitored anesthesia care.

Of the 46 patients, two in the MAC group and one in the GA group were excluded because they underwent additional surgeries (Figure 1).

The remaining patients successfully underwent TURBT using the assigned anesthesia method for their respective group. The median age of the patients was 67 years; 20 (48%) patients were classified as ASA class III, and 23 (53%) patients underwent their second or subsequent surgery. More than half of the patients had bladder tumors smaller than 2 cm. Patient demographics were similar between the groups (Table 1).

### 3.1. Efficacy

The median IAET was shorter in the MAC group than that in the GA group (14 vs. 25 min, *p* < 0.001). The anesthesia time was 15 min shorter in the MAC group than that in the GA group (*p* < 0.001), although the operation time was comparable between the groups. Consequently, the median total OR time was 17 min shorter in the MAC group than that in the GA group. The loss of consciousness time was 108 s in the MAC group and 51 s in the GA group (*p* < 0.001). Awake times were comparable between the groups (Table 2).

The median anesthesia charge for the MAC group (USD 152) was significantly less than that in the GA group (USD 195, *p* < 0.001). The MAC group also had lower total hospital charges and shorter admission days than those in the GA group; however, these differences were not statistically significant (USD 1857 vs. 2099 and 2 vs. 3 days, *p* = 0.091 and 0.890, respectively) (Table 2).

### 3.2. Safety

**Figure 2:**
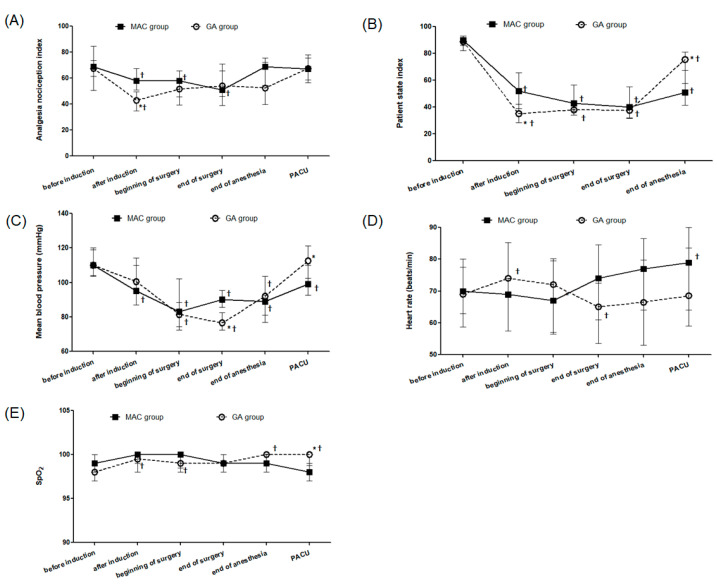
ANI, PSI, and perioperative hemodynamics. (**A**) ANI, (**B**) PSI, (**C**) mean blood pressure, (**D**) heart rate, (**E**) SpO_2_ during TURBT. Values are median (interquartile range). ANI, analgesia nociception index; GA, general anesthesia; MAC, monitored anesthesia care; PACU, postanesthesia care unit; PSI, patient state index; SpO_2_, pulse oximetry; TURBT, transurethral resection of bladder tumor. * Bonferroni-corrected *p* < 0.05, compared to the MAC group. † Bonferroni-corrected *p* < 0.05, compared with the value of baseline in each group.

Data of ANI, PSI, and perioperative hemodynamics are shown in Figure 2.

The ANI value in the MAC group was maintained at the target level (50–70) throughout surgery. After induction, the median ANI value was significantly lower in the GA group than in the MAC group (43 vs. 58, *p* < 0.001) (Figure 2A). The GA group showed a significantly lower mean blood pressure at the end of surgery (77 vs. 90 mmHg, *p* < 0.001), but a higher value at PACU (113 vs. 99 mmHg, *p* = 0.002) than those in the MAC group, indicating that hemodynamic fluctuations were pronounced in the GA group (Figure 2C). The heart rate was comparable between the groups (Figure 2D). The median SpO_2_ was higher at PACU in the GA group (100 vs. 98, *p* = 0.018). However, it remained above 98% throughout the study period in both groups (Figure 2E). The incidence of hypotension during surgery was significantly higher in the GA group (73%) than that in the MAC group (29%) (*p* = 0.004). Seven patients (33%) in the MAC group experienced intraoperative desaturation, and all of them recovered without complications through the jaw-thrust maneuver; none of the patients experienced desaturation in the GA group (*p* = 0.004). The median MOAA/S score at PACU arrival was lower in the MAC group than that in the GA group (4 vs. 5, *p* < 0.001). None of the patients in the MAC group received flumazenil. None of the patients in either group reported nausea at PACU and 6 h postoperatively. The median pain scores at PACU (0 vs. 1, *p* = 0.085) and at 6 h postoperatively (2 vs. 2, *p* = 0.590) were not significantly different between the groups (Table 3).

### 3.3. Feasibility

Surgeons’ satisfaction was “excellent” in all patients in the GA group and 15 patients (71%) in the MAC group. Among the remaining six patients in the MAC group, surgeons’ satisfaction was rated as “good” in three patients (14%); “satisfactory” in one (5%); “poor” in one (5%); and “very poor” in one (5%) (*p* = 0.009). All patients expressed their satisfaction as “excellent” in both groups (Table 3).

## 4. Discussion

In this randomized-controlled study, remimazolam-based MAC guided by ANI monitoring significantly reduced the IAET during TURBT compared to inhalation-based GA, specifically for small bladder tumors. The anesthesia charge was also lower, and the operation was performed safely in all patients without any complications or failure in the MAC group.

The OR is a financial hub and a major source of both revenue and expenses within the hospital, accounting for up to 35−40% of a hospital’s cost and 60−70% of its revenue [7,19,20]. OR efficiency encompasses OR throughput, utilization, and time, with much focus on reducing turnover time and prompt last-second cancellation [8]. Recently, reducing OR occupying time as well as turnover time has gained importance. Improving anesthesia-controlled time-related efficiencies is crucial for maintaining the quality of care and patient safety while optimizing the overall OR performance. TURBT, a high-throughput procedure, is particularly suitable for studying anesthesia-controlled time efficiencies as non-procedural time can approach 50% of total operation time [10]. Additionally, the high prevalence of elderly patients [6] presents challenges in the management of anesthesia for TURBT. In that regard, MAC has the potential to be an optimal choice for TURBT as it offers safe and efficient anesthesia by eliminating the need for mechanical ventilation. Therefore, this study aimed to investigate the efficacy and safety of MAC in patients undergoing TURBT.

In this study, all patients underwent surgery without complications. Although the operation time was comparable between the MAC and GA groups, OR occupying time was significantly shorter in the MAC group. Specifically, the median IAET and total OR times were 11 and 17 min shorter, accounting for approximately 27.5% of total anesthesia time and 38% of the total OR time in the GA group. These reductions could translate into substantial improvements in OR performance and utilization, with reported OR costs ranging from USD 30 to over USD 100 per minute [7]. Anesthesia charges were significantly lower in the MAC group, at 78% of those in the GA group. Total hospital charges were also lower in the MAC group, though not statistically significant, possibly due to the relatively low proportion of anesthesia charges (approximately 10% of the total hospital charge) in TURBT.

Many elderly patients have impaired pulmonary function from conditions like chronic obstructive pulmonary disease or emphysema, complicating anesthesia management. Avoiding mechanical ventilation helps prevent pulmonary complications and improve overall outcomes, especially given the high recurrence rate of bladder tumors and their prevalence in elderly patients [14,21]. Notably, 54% of the patients underwent their second or subsequent TURBT, with one patient undergoing his 9th. Recently, non-intubated thoracic surgery has been introduced to maintain spontaneous breathing without tracheal intubation, aiming to reduce GA-associated side effects, aligning with the context of this study [22].

The MAC group showed significantly better hemodynamic stability, with a lower incidence of hypotension (29% vs. 73%). The median mean blood pressure in the MAC group was higher at the end of surgery (90 vs. 77 mmHg), and lower at PACU arrival (99 vs. 113 mmHg), highlighting the greater degree of hemodynamic fluctuation in the GA group. GA often requires higher anesthetic doses, which can cause hemodynamic instability, leading to complications such as cardiac depression and loss of autonomic reflex [23,24]. Su et al. reported that sedation during percutaneous procedures lowers in-hospital and 30-day mortality, likely due to reduced risks of neurologic deficits, myocardial ischemia, and renal impairment [25]. Additionally, MAC avoids the need for LMA insertion or tracheal intubation, thereby reducing the risk of sympathetic stimulation and pulmonary complications [25,26].

This study confirms the efficacy and safety of remimazolam-based MAC in patients undergoing TURBT for small bladder tumors. Remimazolam, an ultra-short-acting benzodiazepine, allows for rapid onset and recovery due to its short elimination half-life [16]. The onset time of remimazolam is 1–2 min, while that of propofol is less than 1 min [27]. In this study, the GA group showed an onset time of 51 s, and the MAC group showed 108 s, demonstrating similar results. Although the MAC group exhibited a longer loss of consciousness time by 57 s, the IAET, our primary outcome, measured in minutes, was significantly shorter. Therefore, this difference did not appear to be of substantial clinical relevance. Compared to midazolam or propofol, remimazolam provides a shorter awake time, better patient satisfaction, and lower risks of respiratory depression and hypotension [28,29]. Although the MOAA/S score was lower upon PACU arrival in the MAC group, all patients scored 3 or higher, with no differences in recovery time. While surgeons reported lower satisfaction in the MAC group, the operation time tended to be shorter, suggesting minimal impact on surgical performance. One third of patients in the MAC group experienced desaturation events, all of which were effectively and promptly managed with a jaw-thrust maneuver, allowing all procedures to be completed without complications. These results indicate that desaturation events are both preventable and manageable with appropriate drug concentration adjustments, careful patient selection, and early intervention at the onset of hypoventilation. Further studies are needed to improve surgeons’ satisfaction and reduce temporary desaturation events. Therefore, based on our data, remimazolam-based MAC could be a safer alternative to inhalation-based GA in patients undergoing TURBT, specifically for small bladder tumors.

Proper sedation and pain relief while maintaining spontaneous breathing are crucial during MAC, requiring careful dosing of anesthetics and analgesics, with ANI monitoring aiding this process. Studies have reported that ANI monitoring reduces opioid use during GA, aligning with the enhanced recovery after surgery (ERAS) strategy to enhance outcomes [30,31]. The ANI values were maintained within the target level (50–70). However, ANI values were significantly lower in the GA group immediately after induction. This is presumed to be attributed to sympathetic activation after LMA insertion or tracheal intubation. Most patients did not complain of pain or nausea after surgery. All these data indicate that ANI monitoring allows the anesthesia level to be maintained within the objective and quantified anesthesia strategies, effectively preventing both excessive and insufficient medication administration and ultimately promoting ERAS.

The strength of this study lies in being the first study to investigate the impact of remimazolam-based MAC in TURBT. However, there are several limitations in this study. First, blinding was not implemented; therefore, it is recommended that future studies incorporate blinding to a certain degree. Second, this study demonstrated that the MAC effectively reduced anesthesia time and cost. However, these reductions may not have translated into a significant decrease in the total hospital charges, likely because of Korea’s healthcare insurance system [32]. Third, this study did not address the impact of reduced IAET on the quality and efficiency of the hospital as a whole. Only data on the individual costs incurred by patients are presented, as data on the cost of OR time, which is determined by factors such as staff wages and other operational expenses, are lacking. Therefore, further studies are needed to investigate the impact of reducing OR occupying time on OR performance and hospital resource utilization efficiency. Fourth, since this study primarily involved patients with small bladder tumors, the findings of this study should be generalized with caution.

## 5. Conclusions

In conclusion, remimazolam-based MAC reduced the IAET in patients undergoing TURBT, specifically for small bladder tumors. ANI monitoring enables the precise adjustment of anesthetic and analgesic dosages including opioids, while maintaining spontaneous breathing during surgery, which may facilitate ERAS. Additionally, remimazolam-based MAC guided by ANI monitoring is expected to reduce OR time and conserve the medical resources required for mechanical ventilation, ultimately helping to lower healthcare costs and optimize OR efficiency.

## Figures and Tables

**Table 1:** Demographic data.

Variables	Total Cohort (n = 43)	MAC (n = 21)	GA (n = 22)	*p* Value
Age (years)	67 (60–72)	69 (61–75)	63 (58–71)	0.122
Male/female (n)	38/5	20/1	18/4	0.345
Height (cm)	167 (162–172)	167 (162–172)	168 (159–172)	0.846
Weight (kg)	65 (61–74)	66 (62–71)	64 (59–76)	0.942
ASA I/II/III	4/19/20	2/8/11	2/11/9	0.814
Medical history (n)				
Hypertension	17 (40%)	6 (29%)	11 (50%)	0.151
Diabetes	13 (30%)	4 (19%)	9 (41%)	0.119
CAOD	1 (2%)	1 (5%)	0	0.488
COPD	9 (21%)	6 (29%)	3 (14%)	0.281
Current medication (n)				
BB	3 (7%)	1 (5%)	2 (9%)	>0.999
CCB	8 (19%)	4 (19%)	4 (18%)	>0.999
ARB	10 (23%)	3 (14%)	7 (32%)	0.281
ACE inhibitor	2 (5%)	1 (5%)	1 (5%)	>0.999
Statin	24 (56%)	10 (48%)	14 (64%)	0.290
Metformin	8 (19%)	3 (14%)	5 (23%)	0.698
SGLT2 inhibitor	6 (14%)	1 (5%)	5 (23%)	0.185
Aspirin	7 (16%)	3 (14%)	4 (18%)	>0.999
Insulin	2 (5%)	0	2 (9%)	0.488
Synthyroid	3 (7%)	2 (10%)	1 (5%)	0.607
Characteristics of surgery (n)				
Number of repeated TURBT for each patient	2 (1–3)	2 (1–3)	2 (1–3)	0.544
Surgery complexity (simple/complex/highly complex)	24/13/6	12/6/3	12/7/3	>0.999
Number of tumor lesions	1 (1–2)	1 (1–2)	1 (1–2)	0.893
Multiple masses	13 (30%)	6 (29%)	7 (32%)	0.817
Lateral bladder mass	19 (44%)	8 (38%)	11 (50%)	0.432
Maximum size of mass (<2 cm/2–5 cm/>5 cm)	26/11/6	12/7/2	14/4/4	0.419

Values are median (interquartile range) or the number of patients (percentage). ACE inhibitor, angiotensin-converting enzyme inhibitor; ARB, angiotensin receptor blocker; ASA, American Society of Anesthesiologists (ASA) physical status; BB, beta blocker, CAOD, coronary artery occlusive disease; CCB, calcium channel blocker; COPD, chronic obstructive pulmonary disease; GA, general anesthesia; MAC, monitored anesthesia care; SGLT2 inhibitor, sodium-glucose cotransporter 2 inhibitor; TURBT, transurethral resection of bladder tumor.

**Table 2:** Various time durations and cost-related variables during TURBT.

Variables	Total Cohort (n = 43)	MAC (n = 21)	GA (n = 22)	*p* Value
Induction and emergence time (min)	20 (14–25)	14 (12–20)	25 (20–28)	<0.001
Anesthesia time (min)	35 (25–45)	25 (20–35)	40 (30–48)	<0.001
Operation time (min)	11 (7–20)	10 (7–18)	14 (7–21)	0.140
Total operating room time (min)	36 (28–47)	28 (25–37)	45 (34–52)	0.001
Loss of consciousness time (s)	90 (50–120)	108 (90–128)	51 (39–66)	<0.001
Awake time (s)	568 (438–690)	551 (376–735)	581 (450–645)	0.723
Recovery time (min)	30 (30–36)	30 (30–37)	30 (30–33)	0.583
Anesthesia charge (USD)	193 (152–195)	152 (142–172)	195 (193–223)	<0.001
Surgery charge (USD)	915 (803–1019)	835 (773–972)	950 (828–1052)	0.074
Total hospital charge (USD)	1979 (1725–2261)	1857 (1725–2019)	2099 (1737–2397)	0.091
Admission day (d)	2 (2–3)	2 (2–3)	3 (2–3)	0.890

Values are median (interquartile range). GA, general anesthesia; MAC, monitored anesthesia care; TURBT, transurethral resection of bladder tumor.

**Table 3:** Variables related the safety and feasibility.

Variables	Total Cohort (n = 43)	MAC (n = 21)	GA (n = 22)	*p* Value
Hypotension (n)	22 (51%)	6 (29%)	16 (73%)	0.004
Desaturation * (n)	7 (16%)	7 (33%)	0	0.004
Lowest ANI	39 (32–47)	43 (38–50)	35 (30–41)	0.004
MOAA/S score at PACU arrival (3/4/5)	9/11/23	9/6/6	0/5/17	<0.001
MOAA/S score at PACU arrival	5 (4–5)	4 (3–5)	5 (4.8–5)	<0.001
Total doses of drug used during TURBT				
Remifentanil (mcg)	146 (112–200)	153 (124–204)	120 (100–200)	0.111
Propfol (mg)	-	-	80 (70–80)	
Remimazolam (mg)	-	22 (19–30)	-	
Pain intensity				
At PACU	1 (0–1)	0 (0–1)	1 (0–1)	0.085
6 h postoperatively	2 (1–2)	2 (2–2)	2 (1–3)	0.590
PONV intensity				
At PACU	0 (0–0)	0 (0–0)	0 (0–0)	>0.999
6 h postoperatively	0 (0–0)	0 (0–0)	0 (0–0)	>0.999
Patients receiving rescue analgesics (n)				
At PACU	0 (0–0)	0 (0–0)	0 (0–0)	>0.999
0–6 h	0 (0–0)	0 (0–0)	0 (0–1]	0.209
Patients receiving rescue antiemetics (n)				
At PACU	0 (0–0)	0 (0–0)	0 (0–0)	>0.999
0–6 h	0 (0–0)	0 (0–0)	0 (0–0)	>0.999
Patient satisfaction (1/2/3/4/5) ^†^	0/0/0/0/43	0/0/0/0/21	0/0/0/0/22	>0.999
Surgeon satisfaction (1/2/3/4/5) ^†^	1/1/1/3/37	1/1/1/3/15	0/0/0/0/22	0.009

Values are median (interquartile range) or the number of patients (percentage). ANI, analgesia nociception index; GA, general anesthesia; MAC, monitored anesthesia care; MOAA/S score, Modified Observer’s Alertness/Sedation score; PACU, postanesthesia care unit; PONV, postoperative nausea and vomiting; TURBT, transurethral resection of bladder tumor. * All patients recovered without complications through the jaw-thrust maneuver. ^†^ Satisfaction scores were rated using a five-point scale: very poor (1), poor (2), satisfactory (3), good (4), excellent (5).

## Data Availability

The datasets generated during and/or analyzed during the current study are available from the corresponding author on reasonable request.

## Abbreviations

The following abbreviations are used in this manuscript:

ANI	analgesia nociception index
ASA	American Society of Anesthesiologists
BB	beta blocker
CAOD	coronary artery occlusive disease
CCB	calcium channel blocker
COPD	chronic obstructive pulmonary disease
ECG	electrocardiography
ERAS	enhanced recovery after surgery
GA	general anesthesia
IAET	induction and emergence time
LMA	laryngeal mask airway
MAC	monitored anesthesia care
MOAA/S score	Modified Observer’s Alertness/Sedation score
OR	operating room
PACU	postoperative care unit
PONV	postoperative nausea and vomiting
PSI	patient state index
SD	standard deviation
SGLT2 inhibitor	sodium-glucose cotransporter 2 inhibitor
SpO_2_	pulse oximetry
TURBT	transurethral resection of bladder tumor
